# Comparative efficacy of different combinations of acapella, active cycle of breathing technique, and external diaphragmatic pacing in perioperative patients with lung cancer: a randomised controlled trial

**DOI:** 10.1186/s12885-023-10750-4

**Published:** 2023-03-28

**Authors:** Xiaoxue Chen, Chuanzhen Li, Linjuan Zeng, Tiehua Rong, Peng Lin, Qinglin Wang, Zhixing Guo, Hao Long, Jiudi Zhong

**Affiliations:** Department of Thoracic Surgery, State Key Laboratory of Oncology in South China, Collaborative Innovation Center for Cancer Medicine, Sun Yat-sen University Cancer Center, No. 651, Road Dongfengdong, Yuexiu District, Guangzhou, Guangzhou, Guangdong China

**Keywords:** Acapella, Active cycle of breathing technique, External diaphragmatic pacemaker, Functional capacity, Lung cancer

## Abstract

**Background:**

Acapella plus active cycle of breathing technique (ACBT), external diaphragm pacemaker (EDP) plus ACBT have been shown to facilitate the recovery of functional capacity and lung function in patients suffering from airway obstruction but the efficacy in perioperative patients with lung cancer has not been proven.

**Methods:**

We conducted a three-arm, prospective, randomized, assessor-blinded, controlled trial in patients with lung cancer who underwent thoracoscopic lobectomy or segmentectomy in the department of thoracic surgery, China. Patients were randomly assigned (1:1:1) to receive Acapella plus ACBT, EDP plus ACBT, or ACBT group (control group) using SAS software. The primary outcome was functional capacity, measured by the 6-minute walk test (6MWT).

**Results:**

We recruited 363 participants over 17 months: 123 assigned to the Acapella plus ACBT group, 119 to the EDP plus ACBT group, and 121 to the ACBT group. Statistically significant differences were noted for functional capacity between the EDP plus ACBT and control groups at each follow-up time (1-week follow-up: difference = 47.25 m, 95% CI, 31.56–62.93; *P* < 0.001; and 1-month follow-up: difference = 49.72 m, 95% CI, 34.04–65.41; *P* < 0.001), between the Acapella plus ACBT and control groups at postoperative week 1 (difference = 35.23 m, 95% CI, 19.30–51.16; *P* < 0.001) and postoperative month 1 (difference = 34.96 m, 95% CI, 19.03–50.89; *P* < 0.001), and between the EDP plus ACBT and Acapella plus ACBT groups at 1-month follow-up (difference = 14.76 m, 95% CI, 1.34–28.19; *P* = 0.0316).

**Conclusion:**

EDP plus ACBT and Acapella plus ACBT significantly improved functional capacity and lung function in perioperative patients with lung cancer, compared with single-model ACBT, and the effects of EDP plus ACBT were clearly superior to those of other programs.

**Trial registration:**

The study was registered in the clinical trial database (clinicaltrials.gov) on June 4, 2021 (No. NCT04914624).

**Supplementary Information:**

The online version contains supplementary material available at 10.1186/s12885-023-10750-4.

## Background

As a major public health issue worldwide, lung cancer is the second most commonly diagnosed cancer, accounting for approximately 11.7% of all diagnosed cancers, and is the predominant cause of cancer-induced mortality (approximately 1.8 million deaths) [1]. Following the current guidelines of the National Comprehensive Cancer Network, surgical resection is recommended as the dominant treatment modality for lung cancer [2, 3]. However, for patients with lung cancer undergoing surgical resection, traction of the lung and resection of the diseased tissue provoke different levels of surgical stress, which are closely related to the inevitable reduction in lung volume, destruction of the negative pressure of the chest cavity, compression of the lung tissue, and collapse of the alveoli, resulting in impaired functional capacity and lung function [4]. There are available pieces of evidence supporting the associations of perioperative functional capacity or lung function with quality of life and decreased functional capacity and lung function also have been demonstrated as crucial factors to the relatively poor prognosis in postoperative patients with lung cancer [5–7]. Moreover, owing to the preoperative functional capacity, smoking behaviour, and increased respiratory secretions after lung cancer surgery, postoperative pulmonary complications (PPCs), including pulmonary infection, atelectasis, and hypoxemia, occur in up to 59% of patients, and 25% of these are directly associated with functional capacity [8–10]. A large body of evidence has shown that compared with preoperative controls, postoperative patients with lung cancer have impaired functional capacity and lung function [11, 12]. Because functional capacity and lung function are of significant interest due to their value in rehabilitation and prognosis, the number of studies exploring pulmonary rehabilitation is growing [13–15]. Despite some attempts to prepare patients with lung cancer for procedures via education, few efforts have been made to enhance their perioperative functional capacity. In a recent update review, airway clearance techniques (ACTs) were found to improve functional capacity compared with no treatment [16].

The active cycle of breathing technique (ACBT) is an ACT that focuses on the patients controlling their breathing pattern and includes breathing control, 3–4 thoracic expansion exercises, and a forced expiratory technique (huffing) [17]. Within the past decade, single-model ACBT has been widely used globally as usual care in the rehabilitation of perioperative patients with lung cancer. However, based on previous studies and clinical observations, some patients are often reluctant to effectively complete the entire ACBT training, possibly because of pain, weakness, or concern about perturbing the healing process, and they may not rehabilitate to their full potential level of functional capacity immediately after surgery [18]. Furthermore, insufficient evidence is available regarding the superior efficacy of ACBT over that of any other ACT in terms of functional capacity and lung function, and a growing number of studies have suggested the application of ACBT in conjunction with other ACTs [16, 19].

Acapella® (DHD Healthcare, Wampsville, NY, USA; CE approved) is a novel portable ACT for airway clearance that applies positive expiratory pressure with dual actions of pressurised respiration and vibration. Positive expiratory pressure encourages airflow behind secretions and alters sputum rheology (mucus flow), which facilitates secretion clearance without over-inflating the lungs [20]. There has been increasing interest in the implementation of Acapella in conjunction with ACBT as a combined ACT intervention in several therapeutic areas such as bronchiectasis, chronic obstructive pulmonary disease, and stroke, achieving better outcomes of lung function and PPCs than controls [21–23].

External diaphragm pacemaker (EDP, manufactured by Guangzhou Arahelio Biotechnology Co., Ltd., model HLO-GJI3A) stimulates the phrenic nerve through body surface low-frequency pulse electrical stimulation to increase phrenic nerve excitability, phrenic muscle contractility, and diaphragmatic activity, thus achieving the purpose of increasing the effective ventilation volume of alveoli and promoting the rehabilitation of functional capacity. Increasing evidence has suggested that EDP combined with ACBT is beneficial for reducing PPCs in patients with stroke [24]. Combined ACTs, designed to improve the clearance of excessive secretions by more than one mechanism, are frequently recommended to facilitate efficient and effective recovery of functional capacity and lung function in patients with airway obstruction [25]. However, no pilot studies have been conducted to investigate the impact of combined ACT programs Acapella plus ACBT or EDP plus ACBT in perioperative patients with lung cancer, and the efficacy and effectiveness of these programs remain to be confirmed in this population.

To quantify the effects of Acapella plus ACBT or EDP plus ACBT on functional capacity, lung function, and PPCs in postoperative patients with lung cancer, a three-arm, prospective, randomised, assessor-blinded, controlled trial was conducted. The objectives of this study were to (1) determine the efficacy of Acapella plus ACBT or EDP plus ACBT in promoting the rehabilitation of functional capacity, lung function, and PPCs compared with single-model ACBT; and (2) affirm whether one program is superior to the others.

## Methods

### Study design and setting

This randomised (1:1:1), assessor-blinded, controlled trial compared three parallel groups of patients with lung cancer who underwent surgical resection. This study was approved by the Ethics Committee of the hospital, under reference number B2020-173-01, and submitted to the clinical trial database (clinicaltrials.gov; registration number: NCT04914624) on 04/06/2021. All procedures in this study were conducted in accordance with the principles of the Declaration of Helsinki.

### Participants

The participants were patients with lung cancer admitted to the Department of Thoracic Surgery in China. Patients were included if they (1) were aged between 18 and 80 years, (2) were diagnosed with stage I to IIIA non-small cell lung carcinoma subtypes by pathological cytology, (3) were undergoing thoracoscopic lobectomy or segmentectomy, (4) had no history of mental illness and cognitive impairment, (5) had no motor disorders of the limbs and motor contraindications, (6) had a predicted percentage of forced expiratory volume in the first second (FEV1% of predicted) at admission ≥ 80% [26], and (7) provided written informed consent. Detailed exclusion criteria included undergoing pulmonary wedge resection or total pneumonectomy and requiring postoperative mechanical ventilation. Potential participants were also excluded if they were diagnosed with other malignancies, distant metastases, severe organic disease, or illiteracy (inability to understand instructions or training).

### Randomisation and blinding

Participants who satisfied the eligibility criteria were block randomised using SAS software (version 9.4; SAS Institute, Inc.) with a 1:1:1 allocation using random block sizes of 3 and 6. Patients were equally assigned to the Acapella plus ACBT, EDP plus ACBT, or ACBT group. Blinding of participants to the treatment allocation was not possible or desirable, and outcome measures were assessed blindly by investigators who were blinded to the group to which each participant was allocated.

In this study, both research investigators and surgeons verified eligibility. Upon establishing the indication for surgery, a medical research team screened the patients to determine whether they had any health-related conditions that prevented them from participating in the trial. The research investigators then contacted them and arranged an appointment to explain the trial in detail during the scheduled outpatient appointment. Sufficient time was given to patients to inquire about the trial details and decide whether to participate. After written informed consent was obtained following a complete explanation of the study, a multidisciplinary assessment was conducted by physiotherapists, surgeons, and rehabilitation nurses with extensive experience in implementing pulmonary rehabilitation and using these therapies.

### Intervention

After enrolment, patients in all groups participated in a multidisciplinary conventional inpatient rehabilitation program on the day of admission and day before surgery, and the program was standardised using evidence from systematic reviews and mainly included exercise, education, nutritional, respiratory physiotherapy, and psychological counselling [27, 28]. All participants were provided information and instructions regarding perioperative management.

#### Control group

During the control period, participants underwent ACBT treatment, which was standardised using evidence from systematic reviews, comprising 3–5 breath controls, 3–4 thoracic expansion exercises, and 2–3 forced expiratory techniques [29, 30]. The full course of ACBT consisted of five sessions, and participants received training on the day of admission, day before surgery, and postoperative days (POD) 1–3. A comfortable seated or reclined position was assumed by the patients prior to treatment. The training, lasting approximately 20–30 min per session, was delivered by the rehabilitation team in the ward. An instructional booklet was provided on ACBT. The cycle is described in supplementary material Fig. 1, and information on ACBT is detailed in our previously published study [17].

**Fig. 1:**
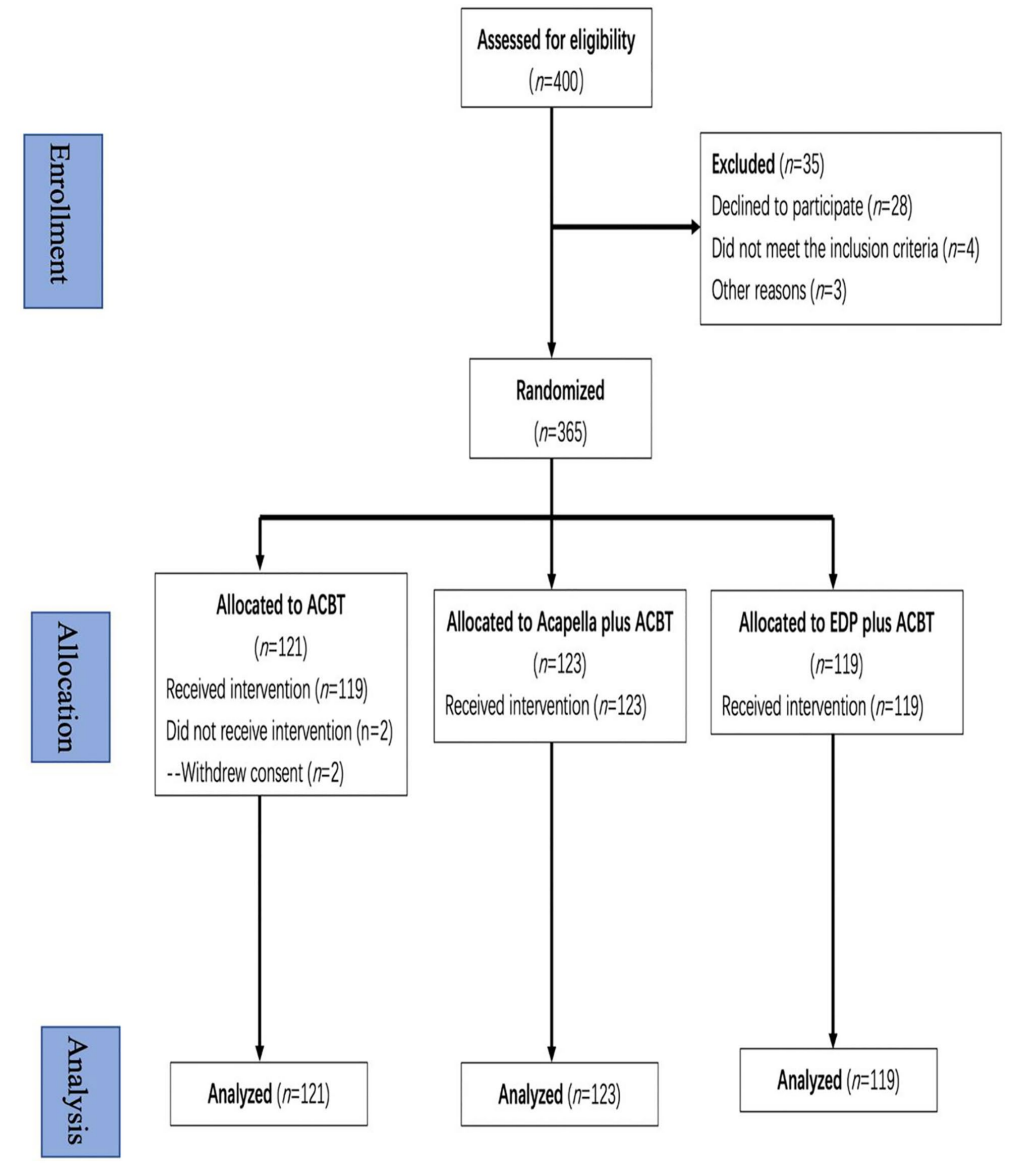
CONSORT 2010 flow diagram for the trial Note: *n*=number; ACBT=active cycle of breathing technique; EDP=external diaphragm pacer.

#### Intervention group

Participants in the Acapella plus ACBT intervention group received a five-session combined ACTs program targeted at facilitating airway clearance on the day of admission, day before surgery, and POD 1–3 in the ward. Initially, the participants were asked to maintain a semi-recumbent position with the abdomen relaxed in a hospital bed, perform 3–4 thoracic expansion exercises with active inspiration, exhale through the Acapella device (frequency/resistance dial set gradually starting from the minimum setting to the maximum setting participants could tolerate) in a 4–6-second timeframe, and control breathing, with eight repetitions of the whole process. Finally, the participants performed the forced expiratory technique (huffing) to clear their airways. Once the participants had been treated for a maximum of 30 min, no longer expectorated sputum, or were too fatigued to continue, the treatment was considered as complete.

In the Acapella plus EDP intervention, all participants and caregivers were instructed by the rehabilitation team on the necessary precautions and received a booklet with detailed instructions. The ACBT procedure in this group was consistent with that in the control group. All patients assumed the supine position, cleaned their neck and chest, and removed surface sebum and sweat before the intervention. The participants underwent EDP treatment, which was provided by physiotherapists, at a pacing frequency of 15 times per minute, pulse frequency of 40 Hz, and stimulation intensities gradually starting from 40 Hz to the intensities that participants could tolerate. Two pairs of electrode sheets were attached by the physiotherapists to the skin at the second intercostal space along the middle clavicular line and the distal one-third of the outer edge of the sternocleidomastoid muscles. The treatment, lasting an average of 30 min, was implemented on the day of admission, day before surgery, and POD 1–3 in the ward.

### Outcomes

The primary outcome was functional capacity in patients with lung cancer, as measured by the 6-minute walk test (6MWT), 1 week preoperatively and 1 month postoperatively. The 6MWT is a standardised test used to predict the prognosis of functional capacity in adult and geriatric patients with cancer [31]. In this study, the test was conducted indoors along a 30-metre hard, flat corridor that had clearly marked turning points for 6 min. The participants walked at their own pace and rested as needed. The better distance walked by the participant in the 6MWT of two tests was recorded in metres (m) and statistically analysed.

The secondary outcome measures were lung function parameters, PPCs, drainage tube removal time, and postoperative hospital stay in patients with lung cancer. Currently, lung function evaluation in clinical practice comprises forced vital capacity (FVC) and FEV1 [32]. The lung function parameters were measured by a trained investigator according to the guidelines of the European Respiratory Society and the American Thoracic Society [33]. Participants performed the tests at least three times at both timepoints 1 week preoperatively and 1 month postoperatively, and the analysis was based on the best reported value. In addition, PPCs were assessed and recorded during the week after surgery by the rehabilitation team based on clinical presentation, laboratory tests, and imaging findings. The incidence of PPCs in all groups was expressed as a percentage.

### Statistical analysis

In this study, PASS 14 (PASS software, Kaysville, Utah, USA; www.ncss.com/software/pass) was used to calculate the sample size of multiple independent groups, setting the two-sided test level at 0.05 and test power at 90%, with three groups. Based on a preliminary study with three groups of 20 patients each (Acapella plus ACBT, EDP plus ACBT, and ACBT groups), the sample size was calculated using an estimate of 13.0 m, 14.5 m, and 20.6 m for the standard deviations (SDs) for the 6MWT distances. Based on these assumptions, a sample size of 28 patients per group was necessary, and an estimated dropout rate of 20% resulted in 34 patients per group.

All analyses were conducted in accordance with the intention-to-treat principle. Continuous variables are expressed as mean (SDs), and categorical variables as frequencies and percentages. Descriptive statistics were used for baseline characteristics and outcomes. Group differences at baseline were reported using one-way analysis of variance, chi-square (χ2) test, or nonparametric test, as appropriate, and the 95% confidence interval (CI) was calculated. Due to the prespecified sequence of rehabilitation training comparisons (ACBT vs. Acapella plus ACBT, ACBT vs. EDP plus ACBT, Acapella plus ACBT vs. EDP plus ACBT; results were interpreted as if from three independent trials, with interpretation for one comparison independent of the outcome of another), no adjustment for multiple comparisons was performed in the study.

For primary and secondary outcome analyses, repeated-measures linear mixed models (LMMs) were conducted to assess the intervention effects. The main effects of group and time and interaction effects between group and time were examined in the LMMs using repeated measurements of the groups at baseline and the two follow-up points (i.e., at the 1-week and 1-month follow-ups), adjusting for baseline demographic and disease-related characteristics (i.e. age, sex, height, weight, education level, smoking amount, smoking years, pathology typing, type of procedure, operative time, anaesthesia time, and bleeding volume) and baseline outcomes of functional capacity and lung function parameters. The R package nlme was used to conduct the LMM analysis. In addition, the R packages ggplot2 (version 3.3.5) and ggprism (version 1.0.3) were used to draw line charts. PPCs were listed by group. The total incidence of PPCs, drainage tube removal time and postoperative hospital stay were compared using the Kruskal-Wallis test. All analyses were conducted using the R software (version 4.1.2). *P* < 0.05 was considered statistically significant.

## Results

### Participant characteristic

Between July 1, 2021, and November 31, 2022, 400 patients with lung cancer were enrolled in the study, and 35 were subsequently excluded because they did not meet the eligibility criteria. Therefore, 365 patients with lung cancer were randomised into groups. Two patients dropped out due to withdrawal of consent, resulting in a dropout rate of 0.5%. According to the intention-to-treat principle, 121 participants in the ACBT group, 123 in the Acapella plus ACBT group, and 119 in the EDP plus ACBT group were finally included in the analysis. The flow of participant enrolment is shown in Fig. 1. The baseline characteristics of participants enrolled in this trial, which were balanced at baseline, are detailed in Table 1. In terms of age, sex, height, and weight, the average baseline values of 6MWT, predicted percentage of FVC (FVC% of predicted), and FEV1% of predicted were within the normal range.

**Table 1:** Baseline characteristics of participant

	Control (*n* = 121)	Acapella plus ACBT (*n* = 123)	EDP plus ACBT (*n* = 119)	*P*-value
Age (yrs), mean (SD)	54.64 (11.19)	56.41 (10.57)	55.93 (10.87)	0.423
Sex, *n* (%)				0.567
Male	57 (47.11)	53 (43.09)	48 (40.34)	
Female	64 (52.89)	70 (56.91)	71 (59.66)	
Height (cm), mean (SD)	162.66 (7.68)	161.47 (7.56)	162.24 (7.91)	0.476
Weight (kg), mean (SD)	61.96 (10.85)	59.61 (10.48)	59.53 (9.57)	0.116
Education status, *n* (%)				0.658
Illiterate and elementary	27 (22.31)	23 (18.70)	25 (21.01)	
Secondary	55 (45.45)	53 (43.09)	50 (42.02)	
Diploma	16 (13.22)	23 (18.70)	14 (11.76)	
University	23 (19.01)	24 (19.51)	30 (25.21)	
Smoking amount, mean (SD)	4.95 (10.44)	4.66 (9.21)	3.29 (7.13)	0.319
Smoking years, mean (SD)	5.62 (11.36)	5.89 (11.29)	4.09 (9.14)	0.370
Pathology typing, *n* (%)				0.122
Squamous cell carcinoma	6 (4.96)	4 (3.25)	0 (0.00)	
Adenocarcinoma	96 (79.34)	94 (76.42)	97 (81.51)	
Other	19 (15.70)	25 (20.33)	22 (18.49)	
Type of procedure, *n* (%)				0.102
Upper left lobectomy	26 (21.49)	25 (20.33)	32 (26.89)	
Lower left lobectomy	25 (20.66)	11 (8.94)	15 (12.61)	
Upper right lobectomy	32 (26.45)	38 (30.89)	39 (32.77)	
Lower right lobectomy	28 (23.14)	33 (26.83)	27 (22.69)	
Middle lobectomy	10 (8.26)	16 (13.01)	6 (5.04)	
Operative time (hrs), mean (SD)	1.81 (0.92)	1.79 (1.01)	1.71 (0.94)	0.683
Anaesthesia time (hrs), mean (SD)	2.54 (1.01)	2.43 (1.07)	2.26 (1.04)	0.104
Bleeding volume (ml), mean (SD)	73.22 (51.87)	64.76 (107.40)	61.09 (61.84)	0.464
6MWT (m), mean (SD)	536.40 (85.76)	535.25 (68.41)	538.23 (69.93)	0.953
FVC% of predicted, mean (SD)	98.40 (16.73)	99.26 (15.83)	99.34 (17.99)	0.891
FEV1% of predicted, mean (SD)	94.21 (17.22)	94.86 (15.63)	94.96 (15.86)	0.926

Note: *n* = number; ACBT = active cycle of breathing technique; EDP = external diaphragm pacemaker; yrs = years; SD = standard deviation; cm = centimetre; kg = kilogram; hrs = hours; ml = millilitres; 6MWT = 6-minute walk test; m = metre; FVC = forced vital capacity; % of predicted = predicted percentage of; FEV1 = forced expiratory volume in 1 s. P < 0.05 was considered statistically significant

### Outcomes

Table 2 summarises the results of between-group comparisons over time for 6MWT and lung function parameters.

**Table 2:** Effects of the intervention on 6-minute walk test and lung function parameters

Follow-up time	ACBT	Acapella plus ACBT		EDP plus ACBT	Acapella plus ACBT vs. ACBT		EDP plus ACBT vs. ACBT		EDP plus ACBT vs. Acapella plus ACBT	
	Mean (SD)				Between-group difference for mean change from baseline (95% CI) ǂ	*P*-value ǂ	Between-group difference for mean change from baseline (95% CI) ǂ	*P*-value ǂ	Between-group difference for mean change from baseline (95% CI) ǂ	*P*-value ǂ
6MWT (m)										
Baseline	536.40 (85.76)		535.25 (68.41)	538.23 (69.93)	- ^a^	-	-	-	-	-
1 week	428.68 (89.90)		462.76 (75.37)	477.75 (63.63)	35.23 (19.30–51.16)	< 0.001	47.25 (31.56–62.93)	< 0.001	12.02 (-1.41–25.44)	0.080
1 month	481.92 (76.63)		515.72 (66.29)	533.46 (64.86)	34.96 (19.03–50.89)	< 0.001	49.72 (34.04–65.41)	< 0.001	14.76 (1.34–28.19)	0.032
FVC% of predicted										
Baseline	98.40 (16.73)		99.26 (15.83)	99.34 (17.99)	-	-	-	-	-	-
1 week	50.68 (12.85)		65.54 (14.52)	70.48 (13.48)	14.00 (10.39–17.6)	< 0.001	18.86 (15.25–22.48)	< 0.001	4.87 (1.38–8.36)	0.007
1 month	63.69 (12.24)		78.46 (13.42)	81.60 (12.94)	13.91 (10.30–17.51)	< 0.001	16.96 (13.35–20.58)	< 0.001	3.06 (-0.43–6.55)	0.087
FEV1% of predicted										
Baseline	94.21 (17.22)		94.86 (15.63)	94.96 (15.86)	-	-	-	-	-	-
1 week	50.10 (13.12)		64.11 (13.99)	69.20 (12.94)	13.35 (9.83–16.87)	< 0.001	18.35 (14.85–21.85)	< 0.001	5.00 (1.65–8.35)	0.004
1 month	62.58 (12.49)		76.42 (13.62)	80.63 (12.23)	13.19 (9.67–16.71)	< 0.001	17.30 (13.80–20.80)	< 0.001	4.11 (0.76–7.46)	0.017

ǂ: Between-group difference for mean change, based on the linear mixed model, adjusted for baseline demographic and disease-related characteristics (i.e. age, sex, height, weight, education level, smoking amount, smoking years, pathology typing, type of procedure, operative time, anaesthesia time, and bleeding volume), and baseline outcomes of functional capacity and lung function parameters, and id as a random effect

a: Not applicable

### Primary study endpoint

**Fig. 2:**
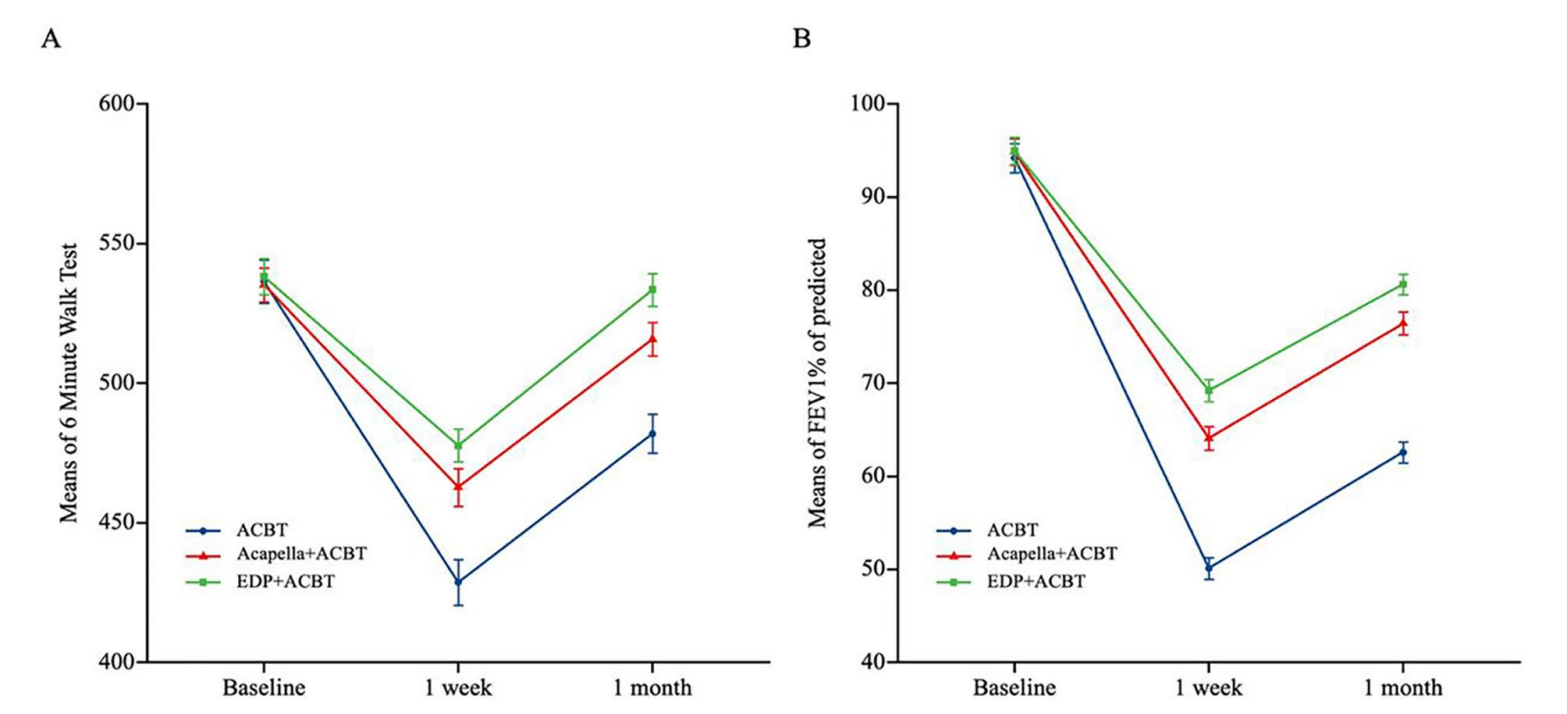
(A) 6-minute walk test, (B) the predicted percentage of forced expiratory volume in the first second Data points indicate means and error bars represent 95% confidence intervals. Note ACBT = active cycle of breathing technique; EDP = external diaphragm pacemaker; FEV1 = forced expiratory volume in one second; % of predicted = the predicted percentage.

At the 1-week follow-up after surgery, the 6MWT of participants was significantly improved in the EDP plus ACBT (difference = 47.25 m; 95% CI, 31.56–62.93; *P* < 0.001) and Acapella plus ACBT groups (difference = 35.23 m; 95% CI, 19.30–51.16; *P* < 0.001) compared with the control group. At the 1-month follow-up, participants in the EDP plus ACBT group had a statistically significantly higher (i.e. better) 6MWT distance than those in the Acapella plus ACBT (difference = 14.76 m; 95% CI, 1.34–28.19; *P* = 0.032) or ACBT (difference = 49.72 m; 95% CI, 34.04–65.41; *P* < 0.001) group. The mean change from baseline in the 6MWT distance at the 1-month follow-up was − 19.53 m in the Acapella plus ACBT group and − 54.49 m in the control group (difference = 34.96 m; 95% CI, 19.03–50.89; *P* < 0.001; Fig. 2), indicating a significant difference between the groups. LMM demonstrated a significant difference between the EDP plus ACBT and Acapella plus ACBT groups at the 1-month follow-up. Additionally, statistically significant differences were noted between the EDP plus ACBT and control groups and between the Acapella plus ACBT and control groups in the 6MWT at each follow-up time.

### Secondary study endpoint

Compared with the control group, participants in the Acapella plus ACBT group had significantly improved FEV1% of predicted at the 1-week (difference = 13.35; 95% CI, 9.83–16.87; *P* < 0.001) and 1-month (difference = 13.19; 95% CI, 9.67–16.71; *P* < 0.001) follow-up; similarly, FEV1% of predicted significantly improved for participants in the EDP plus ACBT group compared with those in the control group at the 1-week (difference = 18.35; 95% CI, 14.85–21.85; *P* < 0.001) and 1-month (difference = 17.30; 95% CI, 13.80–20.80; *P* < 0.001) follow-up. FEV1% of predicted significantly improved for participants in the EDP plus ACBT group compared with those in the Acapella plus ACBT group at the 1-week (difference = 5.00; 95% CI, 1.65–8.35; *P* = 0.004) and 1-month (difference = 4.11; 95% CI, 0.76–7.46; *P* = 0.017) follow-up.

Among the remaining lung function parameters, between-group differences in the FVC% of predicted followed a similar pattern to FEV1% of predicted; FVC% of predicted significantly improved in the Acapella plus ACBT (1-week follow-up: difference = 14.00, 95% CI, 10.39–17.6; *P* < 0.001; and 1-month follow-up: difference = 13.91, 95% CI, 10.30–17.51; *P* < 0.001) and EDP plus ACBT (1-week follow-up: difference = 18.86, 95% CI, 15.25–22.48; *P* < 0.001; and 1-month follow-up: difference = 16.96, 95% CI, 13.35–20.58; *P* < 0.001) groups compared with the control group. Changes in FVC% of predicted over time are seen in Fig. 2 (B) and changes in FEV1% of predicted over time are seen in supplementary material Fig. 2.

Another secondary outcome in this study was the incidence of PPCs in patients with lung cancer, including pulmonary infection, hypoxemia, and atelectasis. In total, 54 (14.88%) of the 363 participants were diagnosed with PPCs, including 28 (23.14%) of 121 in the control group, 16 (13.01%) of 123 in the Acapella plus ACBT group, and 10 (8.40%) of the 119 in the EDP plus ACBT group (Table 3). The total incidence of PPCs significantly reduced for participants in the Acapella plus ACBT group (*P* = 0.040) and EDP plus ACBT group (*P* = 0.002) compared with those in the control group. Moreover, participants randomly assigned to Acapella plus ACBT group had statistically significantly shorter drainage tube removal time (*P* = 0.002) and postoperative hospital stays (*P* < 0.001) than those assigned to the control group. Similarly, the drainage tube removal time (*P* < 0.001) and postoperative hospital stays (*P* < 0.001) for participants in the EDP plus ACBT group were significantly reduced than those in the control group (Table 3).

**Table 3:** Postoperative pulmonary complications, the drainage tube removal time and postoperative hospital stays

	Control (*n* = 121)	Acapella plus ACBT (*n* = 123)	EDP plus ACBT (*n* = 119)	*P-*value			
				Overall	Acapella plus ACBT vs. Control	EDP plus ACBT vs. Control	Acapella plus ACBT vs. EDP plus ACBT
Pulmonary infection, *n* (%)	8 (6.61)	4 (3.25)	3 (2.52)	- a	-	-	-
Hypoxemia, *n* (%)	13 (10.74)	8 (6.50)	5 (4.20)	-	-	-	-
Atelectasis, *n* (%)	7 (5.79)	4 (3.25)	2 (1.68)	-	-	-	-
Total, *n* (%)	28 (23.14)	16(13.01)	10(8.40)	0.005	0.040	0.002	0.248
Drainage tube removal time (hrs)	76 (53.00; 110.00)	66.50 (33.00; 92.50)	52.00 (34.00; 75.50)	< 0.001	0.002	<0.001	0.199
Postoperative hospital stays (days)	4.00 (3.00;6.00)	3.50 (2.00;5.00)	3.00 (2.00; 4.00)	< 0.001	<0.001	< 0.001	0.100

a: Not applicable.

Note: ACBT = active cycle of breathing technique; EDP = external diaphragm pacer; n = number; hrs = hours.

## Discussion

To our knowledge, the present trial is the first to directly compare two combined ACT programs comprising Acapella plus ACBT and EDP plus ACBT, both with one another and with the single-model ACBT program. The results support the hypothesis that preparing perioperative patients for surgical resection of lung cancer with the combined ACTs programs leads to a better functional capacity and lung function after pulmonary surgery compared with the single ACBT program; additionally, the combined ACT programs were safe and effective therapies for the rehabilitation of perioperative patients with lung cancer.

Compared with ACBT, both combined ACT programs significantly improved the 6MWT distance over the 1-week and 1-month postoperative follow-up period. Our finding of short-term improvement in the 6MWT distance is consistent with that reported previously. One study observed a significant improvement in the functional capacity for patients with non-cystic fibrosis bronchiectasis following ACT combined with exercise training, with a mean change (95% CI) in 6MWD of 41 m (95% CI, 19–63) compared with the control group (*P* < 0.05). Another study reported that pulmonary rehabilitation was an effective method in patients with chronic obstructive pulmonary disease for improving the 6MWD distance (*P* < 0.05) [5, 34]. To date, no studies have demonstrated that Acapella plus ACBT or EDP plus ACBT improves functional capacity in patients with lung cancer. Our study is the first to investigate the effects of Acapella plus ACBT or EDP plus ACBT on the functional capacity, which may be in favour of the advanced research and development of combined ACT programs for perioperative patients with lung cancer. However, the postoperative functional capacity did not recover to the preoperative level at 1 month with any of these programs. Hence, the optimum intervention duration remains a matter deserving of future investigation. In addition, a long-term follow-up is warranted to investigate the long-term efficacy of these combined ACT programs.

All three groups in this study showed a reduction in FEV1% of predicted after surgery. In line with our findings, a previous study showed that lung function parameters did not return to baseline levels, possibly because of surgical trauma, wound-related pain, and indwelling of the chest tube [6]. No study has yet determined the recovery time of lung function (FEV1) in patients with lung cancer, and future explorations are required to determine the specific recovery time. Our findings revealed that both Acapella plus ACBT and EDP plus ACBT had significant positive effects on lung function compared with ACBT alone, suggesting that the effects of both combined ACT programs are better, as they facilitate better secretion clearance. However, previous studies have reported that FEV1 remains stable after all ACTs (*P* > 0.05), and this difference may have resulted from differences in study design, sample characteristics, and contexts [22, 35]. Moreover, the association between lung function and functional capacity has been previously reported, and the results of our study were in accordance with the expected improvement in functional capacity [36].

The improvement in FEV1% of predicted in the combined EDP plus ACBT group was significantly better than that in the other groups at both follow-up period. With longer-term follow-ups, an upward trend may persist in the EDP plus ACBT group, which can be explained by the timing of intervention delivery. The study has examined the main effects of group and time and interaction effects between group and time, and the findings of the primary analysis would not be altered. The results of this trial affirmed findings from a previous study, indicating that perioperative patients with lung cancer benefit from EDP plus ACBT with improved lung function [37]. Xu et al. employed a quasi-experimental design, which might have reduced the clinical effect compared with this randomised control trial as a potential selection bias. Nevertheless, the design of this study is more reasonable, which makes the results more persuasive.

In this study, EDP plus ACBT favoured longer walking distances than did the other two therapies. In contrast, a previous study reported no significant difference in the 6MWT distance between breathing training using ACBT (n = 15) and that assisted by EDP plus ACBT (n = 15), which may have resulted from the small sample size [24]. In the present study, the results were more conclusive for the effect of EDP plus ACBT on the 6MWT distance owing to the relatively large sample size. These results accounted for more effective secretion clearance using EDP plus ACBT, resulting in better improvement in lung function and functional capacity.

The incidence rate of PPCs has been estimated to be 15–59% in patients who have undergone lung cancer surgery, which is consistent with the results of this study [10, 38]. In this study, PPCs were rare, and participants could benefit from combined ACTs for preventing PPCs after lung cancer surgery. The results of the study by Li were in agreement with ours, demonstrating that the incidence of PPCs in postoperative patients with lung cancer was 20.6% in the usual care group and 2.9% in the positive vibro-pressure breath training group (*P* = 0.03) [7]. However, Yang et al. reported that the incidence of PPCs did not significantly reduce following ACBT treatment in patients undergoing video-assisted thoracoscopic surgery, and this difference can be attributed to differences in how the intervention was delivered and in populations in terms of conditions and settings [17].

## Limitations

The main limitation of this study was the inability to blind the patients and therapists with regard to treatment allocation; therefore, potential performance bias might have been generated. Our study attempted to reduce the possibility of detection bias, including having assessors of outcomes blind to group allocations. Another limitation is that the baseline 6MWT distances of patients in all groups were > 450 m; therefore, further studies are warranted to explore and investigate the effects of combined ACT programs in the population involving patients walking < 450 m at baseline. The final limitation of this study was its relatively short follow-up period for more analyses of outcomes beyond the 1-month follow-up. Additionally, the long-term effect of the combined ACT program requires further investigation.

## Conclusions

This study found that EDP plus ACBT and Acapella plus ACBT significantly improved functional capacity and lung function in perioperative patients with lung cancer, compared with single-model ACBT, and the effects of EDP plus ACBT were superior to those of Acapella plus ACBT. Thus, both combined ACT programs are effective. These findings encourage healthcare providers to implement combined ACT programs, especially EDP plus ACBT, as part of routine perioperative care.

## Electronic supplementary material

Below is the link to the electronic supplementary material.

Supplementary Material 1

## Data Availability

The datasets used and/or analysed during the current study are available from the corresponding author on request.

## Abbreviations

PPC: Postoperative pulmonary complications
ACT: Airway clearance techniques
ACBT: Active cycle of breathing technique
EDP: External diaphragm pacemaker
FEV1: Forced expiratory volume in the first second
POD: Postoperative days
6MWT: 6-minute walk test
FVC: Postoperative days
SD: Standard deviation
CI: Confidence interval
LMM: Linear mixed model
