# Supplementary material for: Comparative efficacy of different combinations of acapella, active cycle of breathing technique, and external diaphragmatic pacing in perioperative patients with lung cancer: a randomised controlled trial
Source: BMC Cancer. 2023 Mar 28;23:282. doi: 10.1186/s12885-023-10750-4 (PMC10053339; doi:10.1186/s12885-023-10750-4)
Supplement: Supplementary file 1 — Supplementary Material 1 [file 12885_2023_10750_MOESM1_ESM.docx]

**Supplementary material**

Supplementary Figure 1. Cycle of the active cycle of breathing technique

Supplementary Figure 2. Secondary outcome over time for the intervention vs. control groups

Supplementary Figure 1. Cycle of the active cycle of breathing technique

**
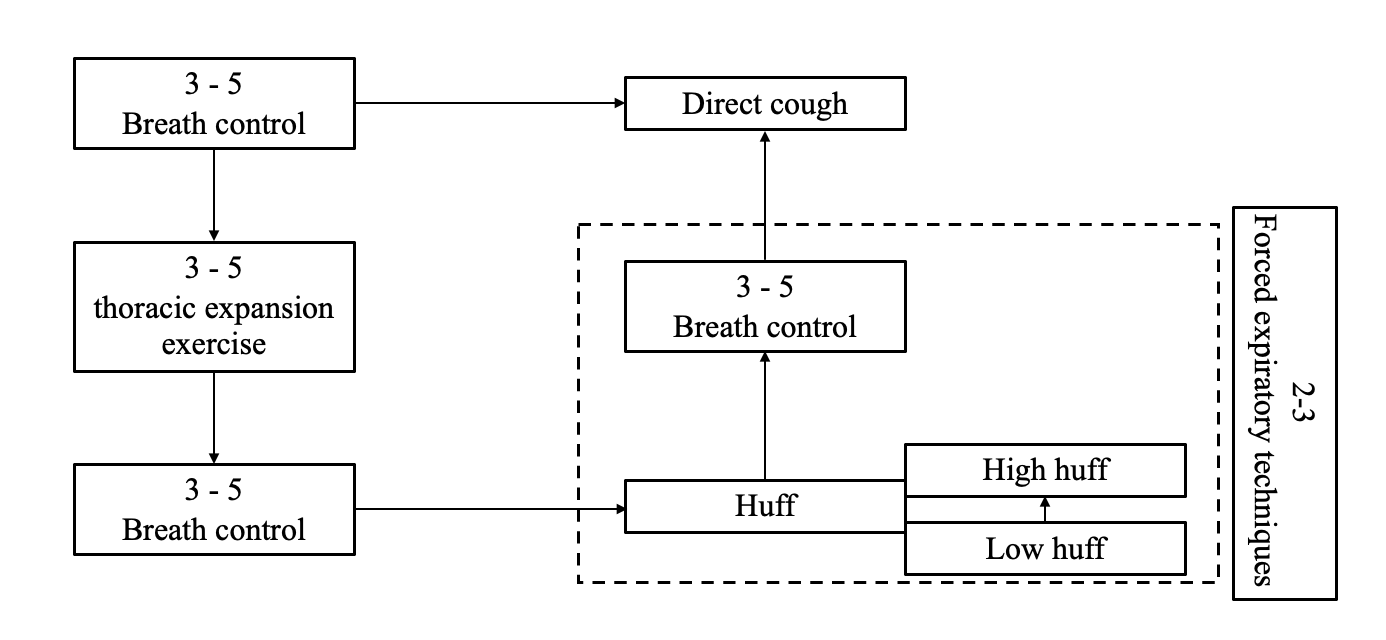
**

Note: ACBT=active cycle of breathing technique.

Supplementary Figure 2. Secondary outcome over time for the intervention vs. control groups


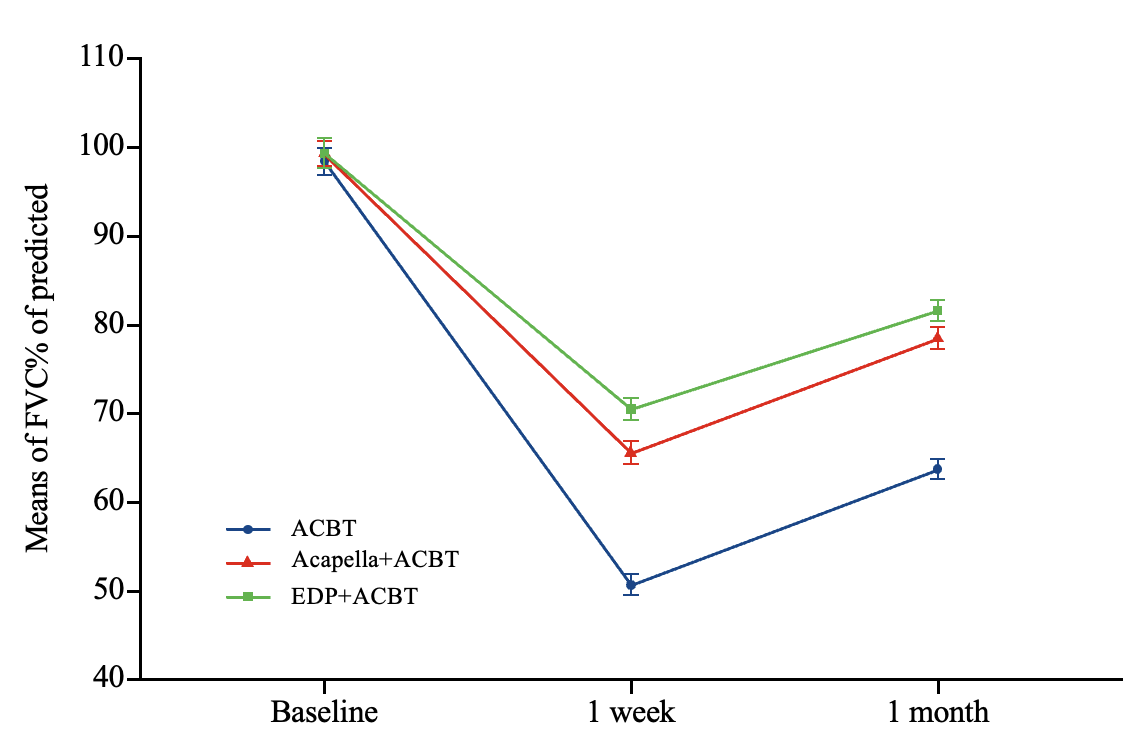


Error bars indicate 95% Confidence intervals.

Note: ACBT=active cycle of breathing technique; EDP=external diaphragm pacer; FVC=forced vital capacity; % of predicted=the predicted percentage.
